# Factors for Rituximab Refractoriness in AQP4‐IgG+ NMOSD: A Cohort Study

**DOI:** 10.1002/acn3.70095

**Published:** 2025-06-10

**Authors:** Mariano Marrodan, María A. Piedrabuena, María A. Zarate, Sofía Rodríguez Murúa, Ezequiel I. Surace, Mauricio F. Farez, Marcela P. Fiol, María C. Ysrraelit, Jorge Correale

**Affiliations:** ^1^ Departamento de Neurología Fleni Buenos Aires Argentina; ^2^ Centro de Investigación en Enfermedades Neuroinmunológicas (CIEN) Fleni Buenos Aires Argentina; ^3^ Laboratorio de Enfermedades Neurodegenerativas Instituto de Neurociencias Fleni‐CONICET (Consejo Nacional de Investigaciones Científicas y Tecnológicas) Buenos Aires Argentina; ^4^ Instituto de Química y Fisicoquímica Biológicas (IQUIFIB) CONICET/Universidad de Buenos Aires Buenos Aires Argentina

**Keywords:** neuromyelitis optica spectrum disorders, NMOSD, rituximab

## Abstract

**Objective:**

Neuromyelitis optica spectrum disorder (NMOSD) is a severe autoimmune condition of the central nervous system (CNS), often associated with aquaporin‐4 antibodies (AQP4‐IgG). Rituximab, a CD20+ B‐cell depleting monoclonal antibody, is widely used as first‐line therapy. However, a subset of patients exhibits treatment refractoriness. Our objective is to investigate factors associated with treatment refractoriness in AQP4‐IgG‐positive NMOSD patients treated with rituximab.

**Methods:**

This retrospective cohort study included 54 AQP4‐IgG‐positive NMOSD patients treated with rituximab between 2006 and 2023. Clinical, imaging, and genetic data were analyzed. Treatment failure was defined as at least one relapse occurring after 6 months of rituximab initiation. Statistical analyses included multivariate analyses of covariance (MANCOVA) and Cox regression to identify independent predictors of treatment failure.

**Results:**

Among the 54 patients (82.5% female, median age 45 years, range: 34–54.5), 12 (22.2%) exhibited rituximab treatment failure. The presence of asymptomatic lesions during follow‐up was significantly associated with treatment failure (*p* = 0.02) and emerged as an independent predictor in MANCOVA (Wilks' Lambda = 0.01, *F* = 20.5, *η* ^2^ = 0.357, *p* < 0.001). These lesions also increased the risk of clinical relapses (HR = 25.9, 95% CI = 3.09–218, *p* < 0.01). Other variables, including age, sex, baseline EDSS, and persistent gadolinium enhancement, were not significantly associated with treatment failure. Genetic analysis of the FCGR3A‐V158F polymorphism did not reveal a significant relationship with treatment outcomes.

**Interpretation:**

Asymptomatic lesions during rituximab treatment are a strong predictor of therapeutic failure in AQP4‐IgG‐positive NMOSD patients. Early identification of these lesions could guide clinicians in optimizing treatment strategies, including transitioning to alternative therapies.

## 1 Introduction

Neuromyelitis optica spectrum disorder (NMOSD) is a chronic autoimmune disease of the central nervous system (CNS), primarily characterized by severe episodes of optic neuritis and transverse myelitis. These features often lead to significant vision loss and motor impairment [1, 2, 3]. A hallmark of NMOSD is the presence of antibodies against aquaporin‐4 (AQP4), a water channel protein highly concentrated on astrocyte end‐feet within the CNS [4, 5]. These pathogenic AQP4‐antibodies (AQP4‐IgG) are detected in a significant proportion of patients (AQP4‐IgG‐positive NMOSD); however, approximately 20%–30% of individuals with NMOSD are seronegative for AQP4‐IgG [6]. Up to 42% of these AQP4‐IgG‐negative NMOSD patients have IgG antibodies against myelin oligodendrocyte glycoprotein (MOG‐IgG) [7, 8].

The binding of AQP4‐IgG to astrocyte AQP4 channels triggers complement activation, followed by granulocyte, eosinophil, and lymphocyte infiltration. This cascade leads to astrocyte damage that results in oligodendrocyte injury, demyelination, and neuronal cell death [9].

Given the relapsing nature of NMOSD and the potential severe neurological sequelae associated with each relapse, chronic immunosuppressive therapy is essential to reduce the number of new episodes and prevent disease disability [10, 11, 12].

Rituximab, a CD20+ B‐cell depleting monoclonal antibody, has gained substantial traction as a first‐line treatment, due to its accessibility and demonstrated efficacy in diverse cohorts [13, 14]. Despite its widespread and overall efficacy, a subset of NMOSD patients does not respond optimally to rituximab. This feature highlights the need to identify predictors of therapeutic failure to minimize delays in initiating appropriate alternative treatments, ensuring the best possible outcomes for these patients.

Recent international therapeutic recommendations have emphasized the need for individualized treatment strategies in NMOSD, incorporating disease severity, serostatus, and regional access to therapies. These guidelines highlight rituximab as a first‐line option in many settings, while also recognizing the growing availability of approved biologics, such as eculizumab, inebilizumab, and satralizumab, which target distinct immunopathogenic mechanisms [15].

This study aims to analyze a cohort of AQP4‐IgG‐positive NMOSD patients to identify clinical, radiological, and genetic factors associated with rituximab treatment failure. Notably, this represents the first investigation in Latin America to assess the presence of FCGR3A polymorphisms previously implicated in suboptimal rituximab response in Asian populations, which can contribute to developing effective therapies earlier in NMOSD patients. Identifying such predictors may facilitate earlier recognition of treatment refractoriness, thereby enabling timely therapeutic adjustments and ultimately contributing to the development of more effective, personalized treatment strategies to optimize clinical outcomes in NMOSD.

## 2 Methods

### 2.1 Patient Selection and Study Design

This retrospective, longitudinal, observational study was conducted on NMOSD patients treated with Rituximab at Fleni, a referral neurological center in Buenos Aires, Argentina, from 2006 to 2023. The neuroimmunology and demyelinating diseases unit at Fleni currently follows 120 NMOSD patients, diagnosed according to the criteria established by Wingerchuk et al. [16] Among these patients, 70 have received rituximab treatment at some point, constituting the study cohort. Only patients with AQP4‐IgG‐positive antibodies were included. AQP4‐IgG was determined using a fixed commercial CBA assay (Euroimmun, Germany), with reported higher specificity and sensitivity than ELISA and IFI [17]. MOG‐IgG was also assessed using a live cell‐based assay at a reference laboratory (Mayo Clinic, USA), and all included patients were MOG‐IgG‐negative [18].

Patients with NMOSD who had not received Rituximab during their disease, those who were seronegative for AQP4 antibodies, patients with positive MOG‐IgG antibodies, or those with other demyelinating diseases (e.g., seronegative recurrent myelitis or chronic recurrent inflammatory optic neuritis), regardless of Rituximab treatment, were excluded from the study. Patients with AQP4‐IgG‐positive NMOSD who had incomplete follow‐up or insufficient information in their clinical records were excluded, as well (*n* = 16).

Data were collected through a comprehensive review of clinical records and structured interviews conducted by trained neurologists in the field. The following variables were analyzed: demographic data including date of birth, date of diagnosis, and sex, as well as clinical factors such as cardiovascular risk factors, disease presentation, and relapse history.

Magnetic resonance imaging (MRI) was performed annually for each patient, focusing on the presence of brain, optic nerve, and spinal cord lesions, and contrast enhancement. All patients underwent comprehensive MRI using a specific protocol designed for multiple sclerosis and demyelinating diseases (3T Discovery MR750; GE Healthcare, Milwaukee, Wisconsin) using a 32‐channel head coil array, including the following: 3D T2‐weighted FLAIR: FOV = 26 × 26 cm; number of slices = 146; voxel resolution = 0.47 × 0.47 × 1.2 mm; TE = 116 ms; TR = 6200 ms; TI = 1710 ms; echo‐train length = 220; acquisition time = 4.18 min; and optimized 3D SWAN‐venule (supplying magnitude and phase) [19]: FOV = 22 × 16 cm; number of slices = 126; voxel resolution = 0.4 × 0.4 × 0.8 mm, with the possibility to reformat to 0.4 × 0.4 × 0.4 mm; TR = 47 ms; TE = 28 ms; flip angle = 8°; echo‐train length = 9; acquisition time = 7.38 min. Both FLAIR and SWAN‐venule sequences were acquired immediately after the intravenous administration of 0.1 mmol/kg of gadolinium‐based contrast agent (gadoterate meglumine, Dotarem; Guerbet, Aulnay‐sous‐Bois, France). Optic nerve imaging protocols included STIR, T2‐weighted, and T1‐weighted sequences, while spinal cord imaging comprised T2‐weighted, STIR, and T1‐weighted sequences. Additionally, postcontrast 3D T1‐weighted sequences were obtained in all cases.

An asymptomatic lesion was defined as the appearance of a cerebral or spinal cord white matter lesion with characteristics suggestive of demyelination or autoimmune origin, in the absence of clinical signs attributable to specific topography [20, 21, 22]. These lesions were identified during scheduled annual MRIs and were prospectively coded during follow‐up and confirmed by two independent neurologists.

Data on the occurrence of relapses, treatment details, and Expanded Disability Status Scale (EDSS) [23] scores before and after treatment were recorded. Relapses were defined as new CNS symptoms and signs lasting longer than 24 h, with or without an associated new lesion on gadolinium‐enhancing MRI, occurring more than 30 days after the initiation of Rituximab treatment [24]. The severity of relapses was assessed clinically and documented in medical records. However, due to the retrospective design of the study and heterogeneity in the severity scoring systems employed across different time points standardized, quantitative severity measures were not uniformly available. Notwithstanding, all relapses required therapeutic interventions, either intravenous methylprednisolone (1 g/day for 3–5 days) or plasmapheresis and/or hospitalization, thereby indicating that these episodes were at least of moderate clinical severity. Given the lack of consensus on the definition of therapeutic failure in NMOSD, a pragmatic criterion was applied: therapeutic failure was defined as at least one clinical relapse after more than 6 months of Rituximab treatment. This approach aligns with clinical practice, where treatment is typically altered when patients experience relapses.

Comprehensive data on rituximab administration, including initiation date, dosing regimen, mean infusion interval, treatment duration, and occurrence of infections, were collected. All patients received an induction protocol consisting of two doses of intravenous infusions of 1 g rituximab administered 2 weeks apart. Maintenance therapy followed one of two protocols: either 375 mg/m^2^ or 1 g every 6 months during the first 2 years. After this period, dosing adjustments were made at the discretion of the treating physician with most patients continuing on 375 mg/m^2^ (*N* = 52, out of 54). B cell depletion was monitored using peripheral CD20^+^ lymphocyte counts; however, due to the retrospective nature of the study and inconsistent monitoring practices, confirmation of complete B‐cell depletion could not be ascertained in all cases. Differences in dosing regimens were analyzed between patients with and without failure, but no statistically significant patterns emerged.

### 2.2 Genetic Studies

Previous studies in Asian populations have shown that rituximab treatment failure is associated with polymorphisms in the FCGR3A gene [25]. To investigate whether the same association was present in our cohort, the rs396991 G/T polymorphism in the *FCGR3A* gene (NM_000569.8) was genotyped blindly. Genetic material was available from nine patients who failed rituximab treatment and 11 randomly selected patients who responded to the treatment (control group). The analysis followed the protocol described by Kim et al. [25] Briefly, genomic DNA was extracted from peripheral blood samples using the Wizard Genomic DNA Purification Kit (Promega). PCR amplification was carried out using the following primers: forward 5′‐CTGTGTCTTTCAGGCTGGC‐3′ and reverse 5′‐AAATGACCAGAATAGTTTAATCTCGT‐3′, followed by Sanger sequencing.

### 2.3 Statistical Analysis

Descriptive statistics were first conducted to assess the distribution of demographic, clinical, and radiological variables. Means, medians, and standard deviations were calculated for continuous variables, while frequencies and percentages were used for categorical variables. Group comparisons were performed using *t*‐tests or Mann–Whitney *U* tests for continuous variables, and chi‐square or Fisher's exact tests for categorical variables. A radar chart (FMSB R library) was used to illustrate these comparisons. Multivariate analysis of covariance (MANCOVA) was employed, to identify independent predictors of poor treatment response, adjusting for potential confounders. A multivariate Cox's regression analysis was also performed to assess the risk of developing relapses under Rituximab treatment. Statistical analyses were conducted using SPSS 21 (SPSS Inc., Chicago, IL, USA). Given the retrospective and hypothesis‐generating nature of the study, all statistical analyses were considered exploratory. Consequently, no correction for multiple comparisons was performed, and the results should be interpreted with appropriate caution.

The study adheres to ethical standards for clinical research, ensuring patient confidentiality and welfare, following STROBE guidelines. Informed consent was obtained from all participants, who were provided with detailed information about the study's objectives, procedures, and potential risks. Patients who underwent genetic testing signed an additional informed consent.

## 3 Results

**FIGURE 1:**
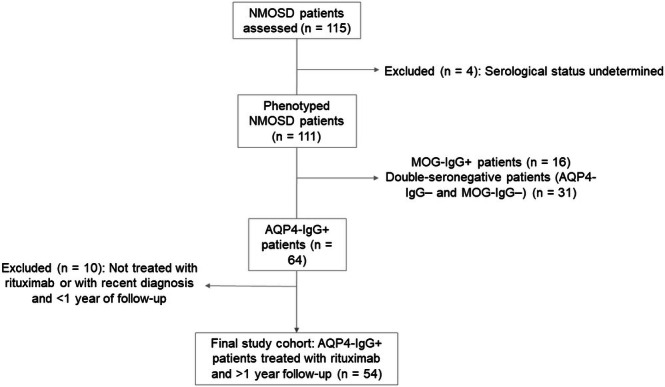
CONSORT diagram illustrating the patient selection process.

**FIGURE 2:**
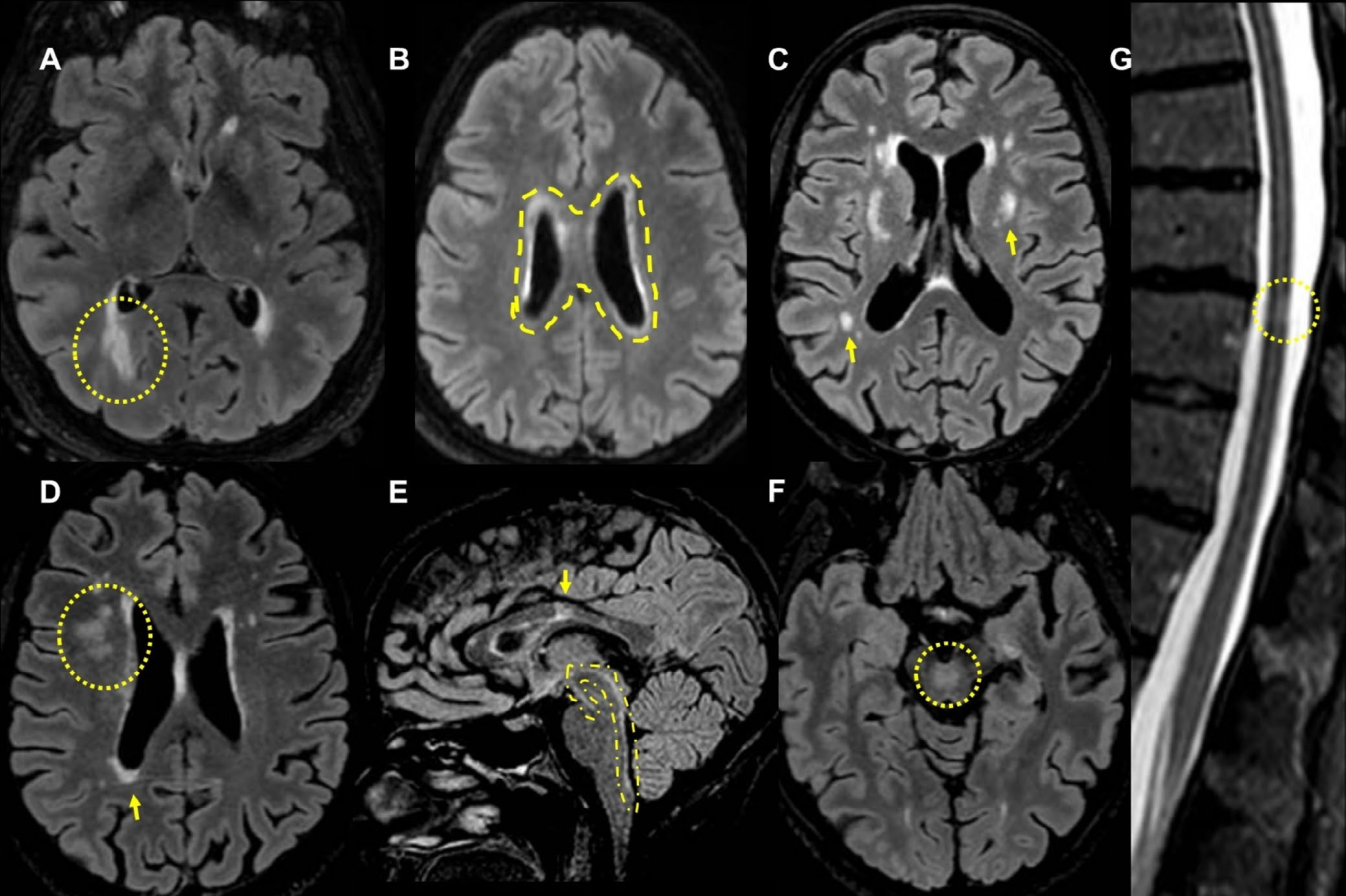
Illustrative examples of asymptomatic lesions observed in patients with AQP4‐positive neuromyelitis optica spectrum disorders. Volumetric FLAIR. (A) Tumefactive periventricular lesion. (B) Periventricular hyperintensity, also referred to as the “pencil sign.” (C) Subcortical demyelinating lesions (yellow arrows). (D) Periventricular and subcortical white matter lesions. (E) Corpus callosum lesions and linear hyperintensity adjacent to the third and fourth ventricles. (F) Periaqueductal hyperintensity. Sagittal STIR. (G) Monosegmental spinal cord lesion.

The study analyzed a cohort of 54 AQP4‐IgG‐positive NMOSD patients, predominantly female (82.5%, *n* = 47), with a median age at rituximab initiation of 45 years (range 34–54.5) and a median EDSS score of 2 (range 1–3) at the start of treatment (Table 1). A CONSORT diagram illustrating patients selection is shown in Figure 1. As expected, the most common initial clinical presentations were longitudinally extensive transverse myelitis (48.1%, *n* = 26) and optic neuritis (46.3%, *n* = 25). A smaller proportion of patients presented with brainstem involvement and/or area postrema syndrome (5.5%, *n* = 3). During the follow‐up period, with a median duration of 52 months (range: 18–92.5), 24.6% (*n* = 14) of patients developed asymptomatic lesions (Table S1), and 21.1% (*n* = 12) exhibited persistent gadolinium enhancement on MRI while receiving rituximab treatment. Asymptomatic lesions were primarily located in the spinal cord and/or in the periventricular or subcortical white matter and exhibited radiological features typical of NMOSD. Lesions in vascular territories or with small vessel disease, or attributable to comorbidities such as hypertension, were excluded. Two neuroimaging experts excluded lesions suggestive of small vessel disease or other unrelated aetiologies by consensus. All asymptomatic lesions were confirmed on follow‐up MRI performed as part of routine clinical care and were anatomically discordant with the clinical status at the time of imaging. A detailed description of the topographies is provided in Table S1 and representative examples of asymptomatic lesions are shown in Figure 2. Twelve patients out of 54 (22.2%) failed rituximab treatment. When comparing patients who responded to treatment with those who failed, some differences emerged (Table 2). Among the 12 patients who failed treatment, a significantly higher percentage (57.1%, *n* = 8) developed asymptomatic lesions during treatment compared to those who responded (14%, *n* = 6; *p* = 0.02) (Table 2). This difference suggests that the presence of asymptomatic lesions may be a significant factor associated with rituximab treatment failure in AQP4‐IgG‐positive NMOSD.

**TABLE 1:** Demographic, clinical, and para‐clinical characteristics of the complete cohort.

Women, *n* (%)	47 (82.5)
Smokers^a^, *n* (%)	13 (22.8)
Relapses in the 2 years prior to rituximab start, median (IQR 1‐3)	1 (1–2)
First clinical symptom, *n* (%)	Myelitis 26 (48.1), Optic neuritis 25 (46.3) Brainstem or area postrema syndrome 3 (5.5)
Age at rituximab initiation, median (IQR 1–3)	45 (34–54.5)
EDSS at rituximab start, median (IQR 1‐3)	2 (1–3)
Previously naïve patients^b^, *n* (%)	24 (42.1)
Asymptomatic lesions during rituximab treatment^c^, *n* (%)	14 (24.6)
Persistent gadolinium‐enhancement during rituximab treatment, *n* (%)	12 (21.1)
Follow up in months, median (IQR 1‐3)	52 (18–92.5)

Abbreviation: EDSS, expanded disability status scale.

^a^ Active smokers and/or ever smokers.

^b^ Refers to patients who had not received prior maintenance immunosuppressive therapy before initiating Rituximab. Remaining patients had been previously treated with agents such as azathioprine or mycophenolate mofetil.

^c^ The values indicate the number and proportion of patients who developed one or more new asymptomatic lesions during treatment.

**TABLE 2:** Comparative analysis of rituximab responders and nonresponders in patients with AQP4‐IgG‐positive NMOSD.

	Failures	No failures	*p*
Women, *n* (%)	13 (92.9)	34 (79.1)	0.2
Smokers^a^, *n* (%)	3 (21.4)	10 (23.3)	0.8
Relapses in the 2 years prior to rituximab start, median (IQR 1–3)	1 (1–1)	1 (1–2)	0.09
Age at rituximab initiation, median (IQR 1–3)	44 (35.25–58.25)	47 (34–54)	0.8
EDSS at rituximab start, median (IQR 1–3)	2 (2–3)	2 (0–3.5)	0.45
Previously naïve patients^b^, *n* (%)	4 (28.6)	20 (46.5)	0.2
Asymptomatic lesions during rituximab treatment^c^, *n* (%)	8 (57.1)	6 (14)	0.02
Persistent gadolinium‐enhancement during rituximab treatment, *n* (%)	2 (14.3)	10 (23.3)	0.7
Follow up in months, median (IQR 1–3)	55.5 (15.25–100.25)	52 (20–92)	0.8

Abbreviation: EDSS, expanded disability status scale.

^a^ Active smokers and/or ever smokers.

^b^ Refers to patients who had not received prior maintenance immunosuppressive therapy before initiating Rituximab. Remaining patients had been previously treated with agents such as azathioprine or mycophenolate mofetil.

^c^ The values indicate the number and proportion of patients who developed one or more new asymptomatic lesions during treatment.

**FIGURE 3:**
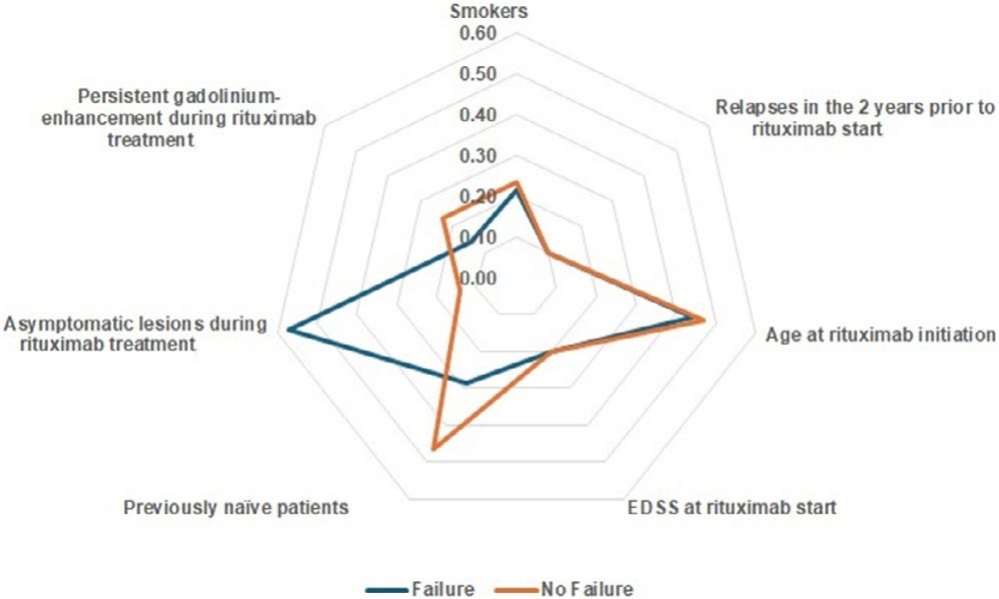
Radar chart comparing clinical and imaging characteristics between patients with and without Rituximab treatment failure. The analysis revealed no statistically significant differences between the two groups in the proportion of smokers, the number of relapses 2 years before rituximab initiation, age at treatment onset, or baseline EDSS. A smaller proportion of treatment‐naïve patients was observed in the failure group (28.6%) compared to the no‐failure group (46.5%), though this difference did not reach statistical significance (*p* > 0.05). Asymptomatic lesions during Rituximab treatment were significantly more frequent in the failure group (57.1%) compared to the no‐failure group (14%) (*p* = 0.02). In contrast, persistent gadolinium enhancement was slightly more common in the no‐failure group (23.3%) than in the failure group (14.3%), but this difference was not statistically significant (*p* > 0.05).

Conversely, other variables such as gender, smoking status, relapses in the 2 years prior to rituximab initiation, age at treatment start, baseline EDSS, prior treatment history, and persistent gadolinium enhancement during treatment did not show statistically significant differences between the two groups (*p* > 0.05 for all comparisons) (Table 2). These differences and similarities are illustrated in Figure 3.

The MANCOVA adjusted for disease duration further emphasized the role of asymptomatic lesions as an independent predictor of inadequate treatment response to rituximab. This analysis revealed that the presence of asymptomatic lesions was strongly associated with treatment failure (Wilks' Lambda = 0.01, *F* = 20.5, *η* ^2^ = 0.357, *p* < 0.001), suggesting that patients with these lesions are significantly more likely to fail rituximab treatment. As expected, other variables such as smoking status, age at rituximab initiation, baseline EDSS, previous treatment history, and persistent gadolinium enhancement did not independently predict treatment failure (*p* > 0.05 for all) (Table 3).

**TABLE 3:** Multivariate analysis of covariance (MANCOVA) to identify independent predictors of inadequate treatment response to rituximab.

Variable	*F*	*η* ^2^	*p*
Smokers^a^	0.35	0.01	0.55
Age at rituximab initiation	1.31	0.03	0.56
EDSS at rituximab start	0.37	0.001	0.84
Previously naïve^b^	0.33	0.009	0.56
Asymptomatic lesions	20	0.35	< 0.001
Persistent gadolinium‐enhancement	0.001	< 0.001	0.98

*Note:* Adjusted by disease duration. Wilks' Lambda = 0.01.

Abbreviation: EDSS, expanded disability status scale.

^a^ Active smokers and/or ever smokers.

^b^ Refers to patients who had not received prior maintenance immunosuppressive therapy before initiating Rituximab. Remaining patients had been previously treated with agents such as azathioprine or mycophenolate mofetil.

Once differences in the cohort were established, the risk of treatment failure was assessed using a multivariate Cox regression analysis. Asymptomatic lesions significantly increased the risk of clinical relapses (HR = 25.9, 95% CI = 3.09–218, *p* < 0.01) compared to persistent gadolinium enhancement (HR = 1.1, 95% CI = 0.2–6.1, *p* = 0.8) and being naïve to rituximab treatment (HR = 0.5, 95% CI = 0.1–2.4, *p* = 0.4). Importantly, asymptomatic lesions were identified prior to the onset of clinical relapses in all cases. The median time interval between lesion detection and subsequent relapse was 6 months (range: 3–11 months), suggesting a temporal and potentially pathophysiological association between subclinical disease and overt clinical exacerbation.

Further, we explore the influence of the FCGR3A‐V158F gene polymorphisms on rituximab efficacy in our cohort [25]. The demographic and disease characteristics did not differ across genotypes. Among the 20 samples analyzed, the distribution of FCGR3A genotypes was as follows: seven homozygous for G (VV), four heterozygous (VF), and nine homozygous for T (FF), corresponding to 35%, 20%, and 45% of the tested patients, respectively. Of the nine patients who failed Rituximab treatment, only three (33%) were homozygous for the V158F polymorphism, and two (22%) were heterozygous. In contrast, among 11 patients who responded to treatment, six (55%) were homozygous for the V158F polymorphism, while two (18%) were heterozygous (Table 4).

**TABLE 4:** FCGR3A‐V158F polymorphism genotyping and core clinical characteristics.

		Failures (*n* = 12)	No failures (*n* = 42)	*p*
FCGR3A‐V158F polymorphisms^a^ *n* = 20 *Codon sequence (Coding Aminoacid)*	AAC (Val), *n* (%)	4 (44.4)	3 (27.2)	0.3
	AAA (Phe), *n* (%)	3 (33.3)	6 (54.5)	
	AAC/AAA (Val/Phe), *n* (%)	2 (22.2)	2 (18.1)	
Clinical characteristics at onset of treatment *n* = 54	Optic Neuritis, *n* (%)	5 (41.6)	21 (50)	0.7
	Myelitis, *n* (%)	6 (50)	19 (45.2)	
	Brainstem or area postrema syndrome, *n* (%)	1 (8.3)	2 (4.6)	

^a^ The values represent the number of patients expressing a particular polymorphism.

Moreover, we analyzed the allele frequency based on an exome database from our population, corresponding to 34%. Interestingly, this percentage increased to 55% in our cohort of 20 patients, with a frequency of 44% among Rituximab nonresponders compared to 64% among responders.

## 4 Discussion

Relapse prevention is the primary goal of NMOSD treatment. According to large case series, more than 40% of patients may become legally blind, and up to 23% may require gait assistance (e.g., cane, wheelchair) within 5 years of diagnosis [2, 26]. Additionally, NMOSD is associated with increased mortality risks, including those related to immunosuppression and infections [27].

Real‐world experience with off‐label therapies like Azathioprine, Mycophenolate Mofetil, Tocilizumab, and Rituximab has been extensively documented. These reports reflect diverse approaches in clinical practice, influenced by drug availability, patient‐specific factors, and local guidelines [28, 29, 30, 31, 32]. Economic and regional disparities significantly impact treatment choices and accessibility. In many low‐ and middle‐income countries, the high cost and limited availability of these therapies pose considerable challenges, with rituximab often being the most accessible first‐line treatment globally. This highlights the importance of considering local healthcare infrastructure and economic constraints when developing treatment protocols and guidelines for NMOSD management [33].

Rituximab is primarily effective through antibody‐dependent cellular cytotoxicity mediated by natural killer (NK) cells via interactions between its Fc fragment and the Fc gamma receptor (FCGR) on NK cells [34]. Its safety and efficacy in preventing relapses have been extensively documented in different case series worldwide and in a clinical trial conducted in Japan [13]. Rituximab has been shown to prevent relapses in more than 60% and up to 88% of cases, even in the long term [24, 35, 36, 37]. However, to the best of our knowledge, this study is the first to specifically analyze relapse risk factors in AQP4‐IgG‐positive NMOSD patients treated with rituximab.

Our findings indicate that the presence of asymptomatic lesions during follow‐up is a primary predictor of subsequent relapses, likely reflecting suboptimal efficacy of rituximab in these cases [38, 39]. Importantly, all asymptomatic lesions observed in our cohort displayed imaging characteristics consistent with NMOSD, such as longitudinally extensive spinal cord lesions and pencil‐thin periventricular hyperintensities [20, 21, 22]. In contrast to previously reported findings, where only 3.4% of clinically stable AQP4‐IgG‐positive NMOSD patients developed new, nonspecific, small (< 6 mm), deep white matter lesions during long‐term follow‐up, our patients showed radiological findings more typical of NMOSD pathology [22]. These findings suggest that asymptomatic lesions in our study likely reflect subclinical disease activity rather than incidental findings unrelated to NMOSD.

Previous studies have shown that female sex, younger age, Afro‐American ethnicity, and coexisting autoimmune disorders are associated with an increased relapse rate. However, these findings were not reflected in our cohort, which was demographically homogeneous with respect to age, race, and ethnicity compared to others [2, 10, 40]. Additionally, persistent gadolinium enhancement, previously identified as a relapse predictor [41], was not associated with increased relapse rates in our study.

In prior studies of Asian patients, a polymorphic variant in the FCGR3A gene (rs396991 G/T), resulting in a valine‐to‐phenylalanine substitution at codon 158 (V158F), was associated with rituximab treatment failure [25]. However, in our cohort, we could not confirm this association, possibly due to the small sample size or due to differences in genetic background.

The presence of asymptomatic lesions during follow‐up was the main finding associated with Rituximab treatment failure. These findings underscore the importance of monitoring asymptomatic lesions in patients undergoing rituximab therapy for AQP4‐IgG‐positive NMOSD. The presence of such lesions could serve as an early marker of suboptimal response to rituximab, prompting clinicians to consider alternative therapeutic strategies earlier in the treatment course. This feature is particularly relevant given the recent approval of novel therapeutic agents targeting distinct immune pathways, such as Eculizumab, Ravulizumab, Inebilizumab, and Satralizumab [42, 43, 44, 45].

Despite the strengths of this deeply phenotyped, genetically tested cohort of AQP4‐IgG‐positive NMOSD patients with extended follow‐up, the study has some limitations. First, as a single‐center study, it lacks the epidemiologic and demographic variability seen in larger, multicentre cohorts. Second, its retrospective design precluded the routine genetic testing of the entire cohort and the evaluation of serum or cerebrospinal fluid biomarkers, such as CD19‐CD20, neurofilaments, and specific chemokines, which could provide valuable insights into the treatment response [46, 47, 48]. Finally, as this study was retrospective, Rituximab dosing and administration schedules varied; however, all strategies employed were previously reported as effective.

Further investigations in larger and prospective cohorts are warranted to validate these observations and refine predictive models for treatment outcomes in AQP4‐IgG‐positive NMOSD. Given the limited sample size, our findings should be interpreted with caution. This study should be considered exploratory and hypothesis generating, serving as a foundation for future prospective validations in larger, multicenter cohorts.

## Author Contributions

M.M. and J.C. contributed to the conception and design of the manuscript. M.M., M.A.Z., M.A.P., S.R.M., E.I.S., M.F.F., M.C.Y., and M.P.F. contributed to the acquisition and analysis of the data. M.M., S.R.M., E.I.S., and J.C. contributed to drafting a significant portion of the manuscript and figures.

## Disclosure

Dr. Mariano Marrodan has received fees for educational presentations and/or conference attendance from Merck‐Serono Argentina, Biogen‐Idec Argentina, Novartis Argentina, Gador, AstraZeneca, Raffo, and Roche Argentina. Dr. Maria A. Zarate has nothing to disclose. Dr. Maria A. Piedrabuena has nothing to disclose. Dr. Sofia Rodriguez Murua has nothing to disclose. Dr. Ezequiel I. Surace has nothing to disclose. Dr. Mauricio F. Farez is the director and cofounder of CIEN. Dr. Maria C. Ysrraelit has received reimbursement for developing educational presentations, attendance to advisory boards, and travel/accommodations stipends from Merck‐Serono Argentina, Biogen, Genzyme Argentina, Bayer Inc., Novartis Argentina, TEVA, and Roche Argentina. Dr. Marcela P. Fiol has received fees for educational presentations and/or conference attendance from Merck‐Serono Argentina, Biogen‐Idec Argentina, Genzyme Argentina, Bayer Inc., Novartis Argentina, Roche Argentina, and TEVA. Dr. Jorge Correale has received financial compensation for academic presentations and attended advisory boards from: Biogen, Merck, Novartis, Roche, Bayer, Sanofi‐Genzyme, Gador, Raffo, Bristol Myers Squibb, and Janssen.

## Conflicts of Interest

The authors declare no conflicts of interest.

## Supporting information


**Table S1:** Asymptomatic lesions characteristics.

## Data Availability

Data not provided in the article because of space limitations may be shared (anonymized) at the request of any qualified investigator for purposes of replicating procedures and results. The Principal Author had full access to all the data used in the analyses presented in this manuscript and takes full responsibility for the integrity of the data, the accuracy of the analyses, and the interpretation of the findings. The authors affirm that they had the right to independently analyze and publish any and all data, without influence from the sponsor. The conduct of this research, including data collection, analysis, and reporting, was carried out independently by the authors.

## References

[1] D. M. Wingerchuk , W. F. Hogancamp , P. C. O'Brien , and B. G. Weinshenker , “The Clinical Course of Neuromyelitis Optica (Devic's Syndrome),” Neurology 53, no. 5 (1999): 1107–1114, 10.1212/wnl.53.5.1107. 10496275

[2] J. Kitley , M. I. Leite , I. Nakashima , et al., “Prognostic Factors and Disease Course in Aquaporin‐4 Antibody‐Positive Patients With Neuromyelitis Optica Spectrum Disorder From the United Kingdom and Japan,” Brain 135, no. Pt 6 (2012): 1834–1849, 10.1093/brain/aws109. 22577216

[3] R. Kadish , S. L. Clardy , M. Royston , et al., “Clinical Burden of Relapses in Aquaporin‐4 Immunoglobulin G‐Positive Neuromyelitis Optica Spectrum Disorder: A Single Center Cohort Analysis,” Journal of Neuroimmunology 362 (2022): 577761, 10.1016/j.jneuroim.2021.577761. 34823121

[4] S. Saadoun , P. Waters , B. A. Bell , A. Vincent , A. S. Verkman , and M. C. Papadopoulos , “Intra‐Cerebral Injection of Neuromyelitis Optica Immunoglobulin G and Human Complement Produces Neuromyelitis Optica Lesions in Mice,” Brain 133, no. Pt 2 (2010): 349–361, 10.1093/brain/awp309. 20047900 PMC2822632

[5] V. A. Lennon , T. J. Kryzer , S. J. Pittock , A. S. Verkman , and S. R. Hinson , “IgG Marker of Optic‐Spinal Multiple Sclerosis Binds to the Aquaporin‐4 Water Channel,” Journal of Experimental Medicine 202, no. 4 (2005): 473–477, 10.1084/jem.20050304. 16087714 PMC2212860

[6] A. Uzawa , F. C. Oertel , M. Mori , F. Paul , and S. Kuwabara , “NMOSD and MOGAD: An Evolving Disease Spectrum,” Nature Reviews. Neurology 20, no. 10 (2024): 602–619, 10.1038/s41582-024-01014-1. 39271964

[7] S. Jarius , F. Paul , O. Aktas , et al., “MOG Encephalomyelitis: International Recommendations on Diagnosis and Antibody Testing,” Journal of Neuroinflammation 15, no. 1 (2018): 134, 10.1186/s12974-018-1144-2. 29724224 PMC5932838

[8] S. H. M. Hamid , D. Whittam , K. Mutch , et al., “What Proportion of AQP4‐IgG‐Negative NMO Spectrum Disorder Patients Are MOG‐IgG Positive? A Cross Sectional Study of 132 Patients,” Journal of Neurology 264, no. 10 (2017): 2088–2094, 10.1007/s00415-017-8596-7. 28840314 PMC5617862

[9] E. A. Nagelhus and O. P. Ottersen , “Physiological Roles of Aquaporin‐4 in Brain,” Physiological Reviews 93, no. 4 (2013): 1543–1562, 10.1152/physrev.00011.2013. 24137016 PMC3858210

[10] M. A. Mealy , D. M. Wingerchuk , B. M. Greenberg , and M. Levy , “Epidemiology of Neuromyelitis Optica in the United States: A Multicenter Analysis,” Archives of Neurology 69, no. 9 (2012): 1176–1180, 10.1001/archneurol.2012.314. 22733096

[11] J. Palace , D. Y. Lin , D. Zeng , et al., “Outcome Prediction Models in AQP4‐IgG Positive Neuromyelitis Optica Spectrum Disorders,” Brain 142, no. 5 (2019): 1310–1323, 10.1093/brain/awz054. 30938427 PMC6487334

[12] E. Carnero Contentti and J. Correale , “Neuromyelitis Optica Spectrum Disorders: From Pathophysiology to Therapeutic Strategies,” Journal of Neuroinflammation 18, no. 1 (2021): 208, 10.1186/s12974-021-02249-1. 34530847 PMC8444436

[13] M. Tahara , T. Oeda , K. Okada , et al., “Safety and Efficacy of Rituximab in Neuromyelitis Optica Spectrum Disorders (RIN‐1 Study): A Multicentre, Randomised, Double‐Blind, Placebo‐Controlled Trial,” Lancet Neurology 19, no. 4 (2020): 298–306, 10.1016/S1474-4422(20)30066-1. 32199095

[14] M. Tahara , T. Oeda , K. Okada , et al., “Compassionate Open‐Label Use of Rituximab Following a Randomised Clinical Trial Against Neuromyelitis Optica (RIN‐2 Study): B Cell Monitoring‐Based Administration,” Multiple Sclerosis and Related Disorders 60 (2022): 103730, 10.1016/j.msard.2022.103730. 35287025

[15] T. Kümpfel , K. Giglhuber , O. Aktas , et al., “Update on the Diagnosis and Treatment of Neuromyelitis Optica Spectrum Disorders (NMOSD)—Revised Recommendations of the Neuromyelitis Optica Study Group (NEMOS). Part II: Attack Therapy and Long‐Term Management,” Journal of Neurology 271, no. 1 (2024): 141–176, 10.1007/s00415-023-11910-z. 37676297 PMC10770020

[16] D. M. Wingerchuk , B. Banwell , J. L. Bennett , et al., “International Consensus Diagnostic Criteria for Neuromyelitis Optica Spectrum Disorders,” Neurology 85, no. 2 (2015): 177–189, 10.1212/WNL.0000000000001729. 26092914 PMC4515040

[17] P. Waters , M. Reindl , A. Saiz , et al., “Multicentre Comparison of a Diagnostic Assay: Aquaporin‐4 Antibodies in Neuromyelitis Optica,” Journal of Neurology, Neurosurgery, and Psychiatry 87, no. 9 (2016): 1005–1015, 10.1136/jnnp-2015-312601. 27113605 PMC5013123

[18] S. Jarius and B. Wildemann , “Aquaporin‐4 Antibodies (NMO‐IgG) as a Serological Marker of Neuromyelitis Optica: A Critical Review of the Literature,” Brain Pathology 23, no. 6 (2013): 661–683, 10.1111/bpa.12084. 24118483 PMC8028894

[19] M. I. Gaitán , P. Yañez , M. E. Paday Formenti , et al., “SWAN‐Venule: An Optimized MRI Technique to Detect the Central Vein Sign in MS Plaques,” AJNR. American Journal of Neuroradiology 41, no. 3 (2020): 456–460, 10.3174/ajnr.A6437. 32054616 PMC7077907

[20] K. Y. Wang , J. Chetta , P. Bains , et al., “Spectrum of MRI Brain Lesion Patterns in Neuromyelitis Optica Spectrum Disorder: A Pictorial Review,” British Journal of Radiology 91, no. 1086 (2018): 20170690, 10.1259/bjr.20170690. 29388807 PMC6223278

[21] M. Y. Lee , K. P. Yong , J. W. Hyun , S. H. Kim , S. H. Lee , and H. J. Kim , “Incidence of Interattack Asymptomatic Brain Lesions in NMO Spectrum Disorder,” Neurology 95, no. 23 (2020): e3124–e3128, 10.1212/WNL.0000000000010847. 32928976

[22] E. P. Flanagan , B. G. Weinshenker , K. N. Krecke , and S. J. Pittock , “Asymptomatic Myelitis in Neuromyelitis Optica and Autoimmune Aquaporin‐4 Channelopathy,” Neurology Clinical Practice 5, no. 2 (2015): 175–177, 10.1212/CPJ.0000000000000104. 26137424 PMC4404285

[23] J. F. Kurtzke , “Rating Neurologic Impairment in Multiple Sclerosis: An Expanded Disability Status Scale (EDSS),” Neurology 33, no. 11 (1983): 1444–1452, 10.1212/wnl.33.11.1444. 6685237

[24] M. A. Mealy , D. M. Wingerchuk , J. Palace , B. M. Greenberg , and M. Levy , “Comparison of Relapse and Treatment Failure Rates Among Patients With Neuromyelitis Optica: Multicenter Study of Treatment Efficacy,” JAMA Neurology 71, no. 3 (2014): 324–330, 10.1001/jamaneurol.2013.5699. 24445513

[25] S. H. Kim , I. H. Jeong , J. W. Hyun , et al., “Treatment Outcomes With Rituximab in 100 Patients With Neuromyelitis Optica: Influence of FCGR3A Polymorphisms on the Therapeutic Response to Rituximab,” JAMA Neurology 72, no. 9 (2015): 989–995, 10.1001/jamaneurol.2015.1276. 26167726

[26] Y. Jiao , J. P. Fryer , V. A. Lennon , et al., “Updated Estimate of AQP4‐IgG Serostatus and Disability Outcome in Neuromyelitis Optica,” Neurology 81, no. 14 (2013): 1197–1204, 10.1212/WNL.0b013e3182a6cb5c. 23997151 PMC3795610

[27] A. Francis , E. Gibbons , J. Yu , et al., “Characterizing Mortality in Patients With AQP4‐Ab+ Neuromyelitis Optica Spectrum Disorder,” Annals of Clinical Translational Neurology 11, no. 7 (2024): 1942–1947, 10.1002/acn3.52092. 38884180 PMC11251462

[28] R. A. Kessler , M. A. Mealy , and M. Levy , “Treatment of Neuromyelitis Optica Spectrum Disorder: Acute, Preventive, and Symptomatic,” Current Treatment Options in Neurology 18, no. 1 (2016): 2, 10.1007/s11940-015-0387-9. 26705758 PMC4807395

[29] C. Trebst , S. Jarius , A. Berthele , et al., “Update on the Diagnosis and Treatment of Neuromyelitis Optica: Recommendations of the Neuromyelitis Optica Study Group (NEMOS),” Journal of Neurology 261, no. 1 (2014): 1–16, 10.1007/s00415-013-7169-7. PMC3895189 24272588

[30] D. J. Kimbrough , K. Fujihara , A. Jacob , et al., “Treatment of Neuromyelitis Optica: Review and Recommendations,” Multiple Sclerosis and Related Disorders 1, no. 4 (2012): 180–187, 10.1016/j.msard.2012.06.002. 24555176 PMC3926208

[31] J. Sellner , M. Boggild , M. Clanet , et al., “EFNS Guidelines on Diagnosis and Management of Neuromyelitis Optica,” European Journal of Neurology 17, no. 8 (2010): 1019–1032, 10.1111/j.1468-1331.2010.03066.x. 20528913

[32] M. C. Papadopoulos , J. L. Bennett , and A. S. Verkman , “Treatment of Neuromyelitis Optica: State‐Of‐The‐Art and Emerging Therapies,” Nature Reviews. Neurology 10, no. 9 (2014): 493–506, 10.1038/nrneurol.2014.141. 25112508 PMC4229040

[33] E. Carnero Contentti , J. I. Rojas , E. Cristiano , et al., “Latin American Consensus Recommendations for Management and Treatment of Neuromyelitis Optica Spectrum Disorders in Clinical Practice,” Multiple Sclerosis and Related Disorders 45 (2020): 102428, 10.1016/j.msard.2020.102428. 32763842

[34] P. Bruhns , B. Iannascoli , P. England , et al., “Specificity and Affinity of Human Fcgamma Receptors and Their Polymorphic Variants for Human IgG Subclasses,” Blood 113, no. 16 (2009): 3716–3725, 10.1182/blood-2008-09-179754. 19018092

[35] Y. Wang , H. Chang , X. Zhang , and L. Yin , “Efficacy of Rituximab in the Treatment of Neuromyelitis Optica Spectrum Disorders: An Update Systematic Review and Meta ‐Analysis,” Multiple Sclerosis and Related Disorders 50 (2021): 102843, 10.1016/j.msard.2021.102843. 33609924

[36] M. T. G. Hayes , R. J. Adam , P. A. McCombe , M. Walsh , and S. Blum , “Long‐Term Efficacy and Safety of Rituximab in the Treatment of Neuromyelitis Optica Spectrum Disorder,” Multiple Sclerosis Journal—Experimental, Translational and Clinical 10, no. 2 (2024): 20552173241257876, 10.1177/20552173241257876. 38807849 PMC11131406

[37] F. Gao , B. Chai , C. Gu , et al., “Effectiveness of Rituximab in Neuromyelitis Optica: A Meta‐Analysis,” BMC Neurology 19, no. 1 (2019): 36, 10.1186/s12883-019-1261-2. 30841862 PMC6402122

[38] N. Collongues , P. Cabre , R. Marignier , et al., “A Benign Form of Neuromyelitis Optica: Does It Exist?,” Archives of Neurology 68, no. 7 (2011): 918–924, 10.1001/archneurol.2011.127. 21747032

[39] V. Camera , L. Holm‐Mercer , A. A. H. Ali , et al., “Frequency of New Silent MRI Lesions in Myelin Oligodendrocyte Glycoprotein Antibody Disease and Aquaporin‐4 Antibody Neuromyelitis Optica Spectrum Disorder,” JAMA Network Open 4, no. 12 (2021): e2137833, 10.1001/jamanetworkopen.2021.37833. 34878547 PMC8655599

[40] X. Ma , A. G. Kermode , X. Hu , and W. Qiu , “Risk of Relapse in Patients With Neuromyelitis Optica Spectrum Disorder: Recognition and Preventive Strategy,” Multiple Sclerosis and Related Disorders 46 (2020): 102522, 10.1016/j.msard.2020.102522. 33007726

[41] G. Orman , K. Y. Wang , Y. Pekcevik , et al., “Enhancing Brain Lesions During Acute Optic Neuritis and/or Longitudinally Extensive Transverse Myelitis May Portend a Higher Relapse Rate in Neuromyelitis Optica Spectrum Disorders,” American Journal of Neuroradiology 38, no. 5 (2017): 949–953, 10.3174/ajnr.A5141. 28302609 PMC5433909

[42] S. J. Pittock , A. Berthele , K. Fujihara , et al., “Eculizumab in Aquaporin‐4‐Positive Neuromyelitis Optica Spectrum Disorder,” New England Journal of Medicine 381, no. 7 (2019): 614–625, 10.1056/NEJMoa1900866. 31050279

[43] B. A. C. Cree , H. J. Kim , B. G. Weinshenker , et al., “Safety and Efficacy of Inebilizumab for the Treatment of Neuromyelitis Optica Spectrum Disorder: End‐Of‐Study Results From the Open‐Label Period of the N‐MOmentum Trial,” Lancet Neurology 23, no. 6 (2024): 588–602, 10.1016/S1474-4422(24)00077-2. 38760098

[44] T. Yamamura , I. Kleiter , K. Fujihara , et al., “Trial of Satralizumab in Neuromyelitis Optica Spectrum Disorder,” New England Journal of Medicine 381, no. 22 (2019): 2114–2124, 10.1056/NEJMoa1901747. 31774956

[45] S. J. Pittock , M. Barnett , J. L. Bennett , et al., “Ravulizumab in Aquaporin‐4‐Positive Neuromyelitis Optica Spectrum Disorder,” Annals of Neurology 93, no. 6 (2023): 1053–1068, 10.1002/ana.26626. 36866852

[46] R. E. Rodin and T. Chitnis , “Soluble Biomarkers for Neuromyelitis Optica Spectrum Disorders: A Mini Review,” Frontiers in Neurology 15 (2024): 1415535, 10.3389/fneur.2024.1415535. 38817544 PMC11137173

[47] X. Chang , W. Huang , L. Wang , et al., “Serum Neurofilament Light and GFAP Are Associated With Disease Severity in Inflammatory Disorders With Aquaporin‐4 or Myelin Oligodendrocyte Glycoprotein Antibodies,” Frontiers in Immunology 12 (2021): 647618, 10.3389/fimmu.2021.647618. 33796113 PMC8008082

[48] O. Aktas , M. A. Smith , W. A. Rees , et al., “Serum Glial Fibrillary Acidic Protein: A Neuromyelitis Optica Spectrum Disorder Biomarker,” Annals of Neurology 89, no. 5 (2021): 895–910, 10.1002/ana.26067. 33724534 PMC8252046

