# Supplementary material for: Factors for Rituximab Refractoriness in AQP4‐IgG+ NMOSD: A Cohort Study
Source: Ann Clin Transl Neurol. 2025 Jun 10;12(8):1566–74. doi: 10.1002/acn3.70095 (PMC12343303; doi:10.1002/acn3.70095)
Supplement: Supplementary file 1 — Table S1: Asymptomatic lesions characteristics. [file ACN3-12-1566-s001.docx]

Supplementary table 1. Asymptomatic lesions characteristics

| Patient No. | Lesion topography | Failure to rituximab | Months to Clinical Relapse |
| --- | --- | --- | --- |
| 1 | Brain, subcortical | Yes | 5 |
| 2 | Brain, corpus callosum | Yes | 11 |
| 3 | Optic Nerve | Yes | 9 |
| 4 | Spinal cord, thoracic | Yes | 4 |
| 5 | Brain, subcortical | Yes | 6 |
| 6 | Brain, subcortical | Yes | 3 |
| 7 | Brain, brainstem | Yes | 7 |
| 8 | Brain, periventricular | Yes | 10 |
| 9 | Brain, subcortical | No | - |
| 10 | Brain, brainstem | No | - |
| 11 | Brain, subcortical | No | - |
| 12 | Brain, subcortical | No | - |
| 13 | Brain, periventricular | No | - |
| 14 | Brain, periventricular | No | - |
